# Significance of ^18^ F-FDG PET and immunohistochemical GLUT-1 expression for cardiac myxoma

**DOI:** 10.1186/1746-1596-9-117

**Published:** 2014-06-16

**Authors:** Yukio Okazaki, Sohsuke Yamada, Shohei Kitada, Iwao Matsunaga, Eijirou Nogami, Teruo Watanabe, Yasuyuki Sasaguri, Yutaka Honma, Tsuyoshi Itou

**Affiliations:** 1 Department of Cardiovascular Surgery, Fukuoka Wajiro Hospital, Fukuoka, Japan; 2 Department of Pathology and Cell Biology, School of Medicine, University of Occupational and Environmental Health, 1-1 Iseigaoka, Yahatanishi-ku, Kitakyushu 807-8555, Japan; 3 Department of Laboratory of Pathology, Fukuoka Wajiro Hospital, Fukuoka, Japan; 4 Department of Nuclear Medicine, Fukuoka Wajiro Hospital, Fukuoka, Japan

**Keywords:** Cardiac myxoma, ^18^ F-FDG PET, Immunohistochemistry, GLUT-1

## Abstract

Cardiac tumours are relatively rare and are difficult to diagnose merely with imaging techniques. We demonstrated an unusual case of left atrial myxoma, displaying the successful detection by positron emission tomography using 2-deoxy-2-[^18^ F] fluoro-D-glucose (^18^ F-FDG PET), correlated closely to more intense and enhanced immunoreactivity with glucose transporter-1 (GLUT-1) in a substantial number of cardiac myxoma cells. Further prospective studies are needed to validate the significance of ^18^ F-FDG PET findings for cardiac myxoma and the association with immunohistochemical GLUT-1 expression in its tumour cells, after collecting and investigating a larger number of surgical cases examined with both of them.

**Virtual slides:**

The virtual slide(s) for this article can be found here: http://www.diagnosticpathology.diagnomx.eu/vs/2991481941253449

## Letter to the editor

The imaging technique of positron emission tomography with 2-deoxy-2-[^18^ F] fluoro-D-glucose (^18^ F-FDG PET) is based on the early observations by Warburg, that neoplastic (especially, malignant) cells accumulate more glucose than non-neoplastic cells do, as a result of high rate of glycolytic catabolism rather than citric acid cycle catabolism [1]. ^18^ F-FDG PET has thus been increasingly performed in the diagnosis, pre-operative cancer staging, or follow-up post-treatment examination of many types of malignancy, whereas few studies have been reported regarding the utility of ^18^ F-FDG PET in intracardiac tumours [2-4]. Herein we reported an unusual case of left atrial myxoma, showing the successful detection by its technique, correlated closely to greater immunoreactivity with glucose transporter-1 (GLUT-1) in a larger number of cardiac myxoma cells.

**Figure 1:**
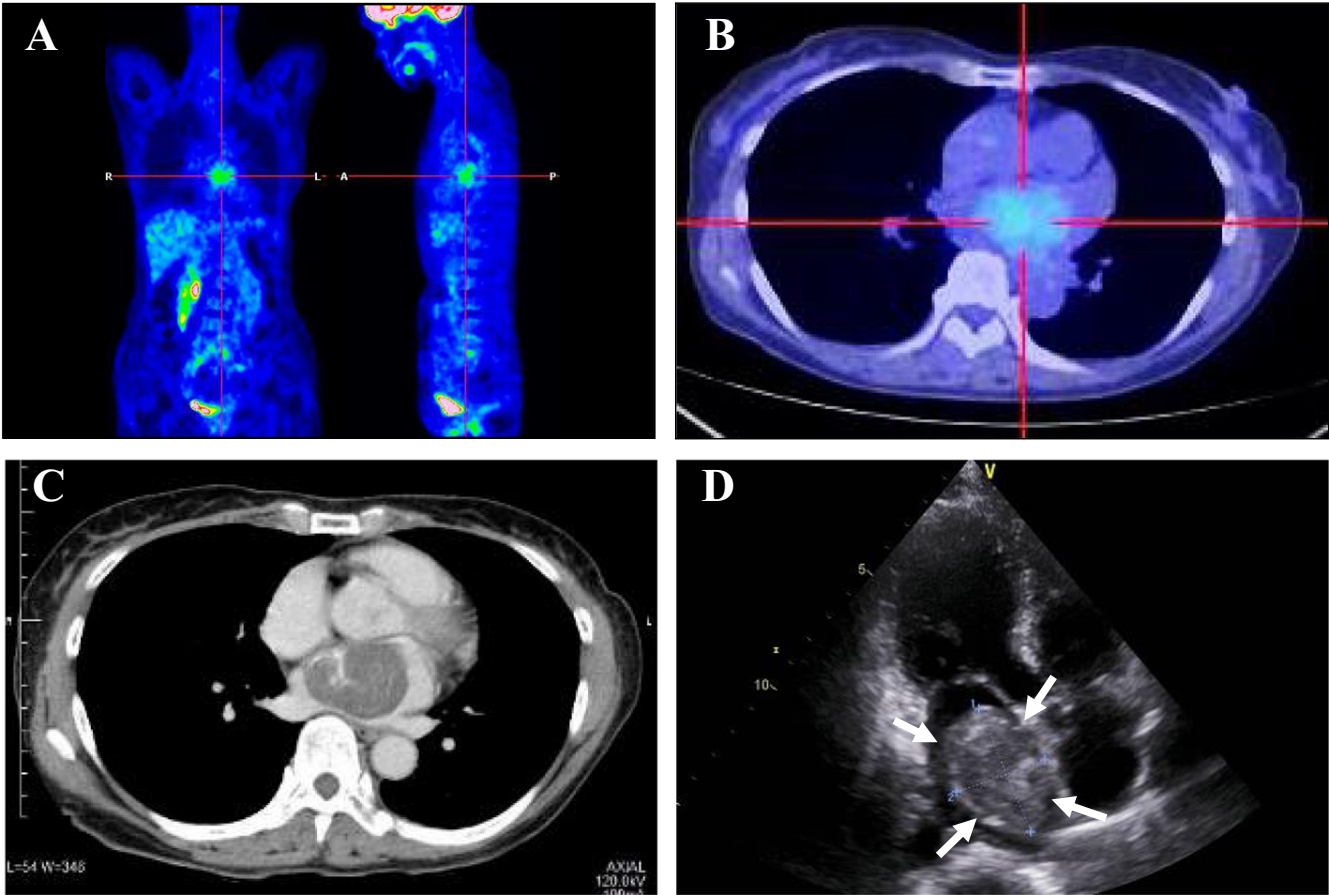
**The findings of**^**18**^ **F-FDG PET/CT and echocardiography at surgery of the resected cardiac myxoma. (A–C)** Coregistered ^18^ F-FDG PET/CT images in coronal **(A)** and axial **(B)** plane demonstrated a large and mildly to moderately hypermetabolic area in the left atrium (maximal SUV: 3.0), which corresponded to a hypodensisty mass lesion on chest CT **(C)**, measuring 46 × 32 mm in diameter. **(D)** Subsequent transthoracic echocardiography revealed a pedunculated mass originating from the interatrial septum (arrows).

**Figure 2:**
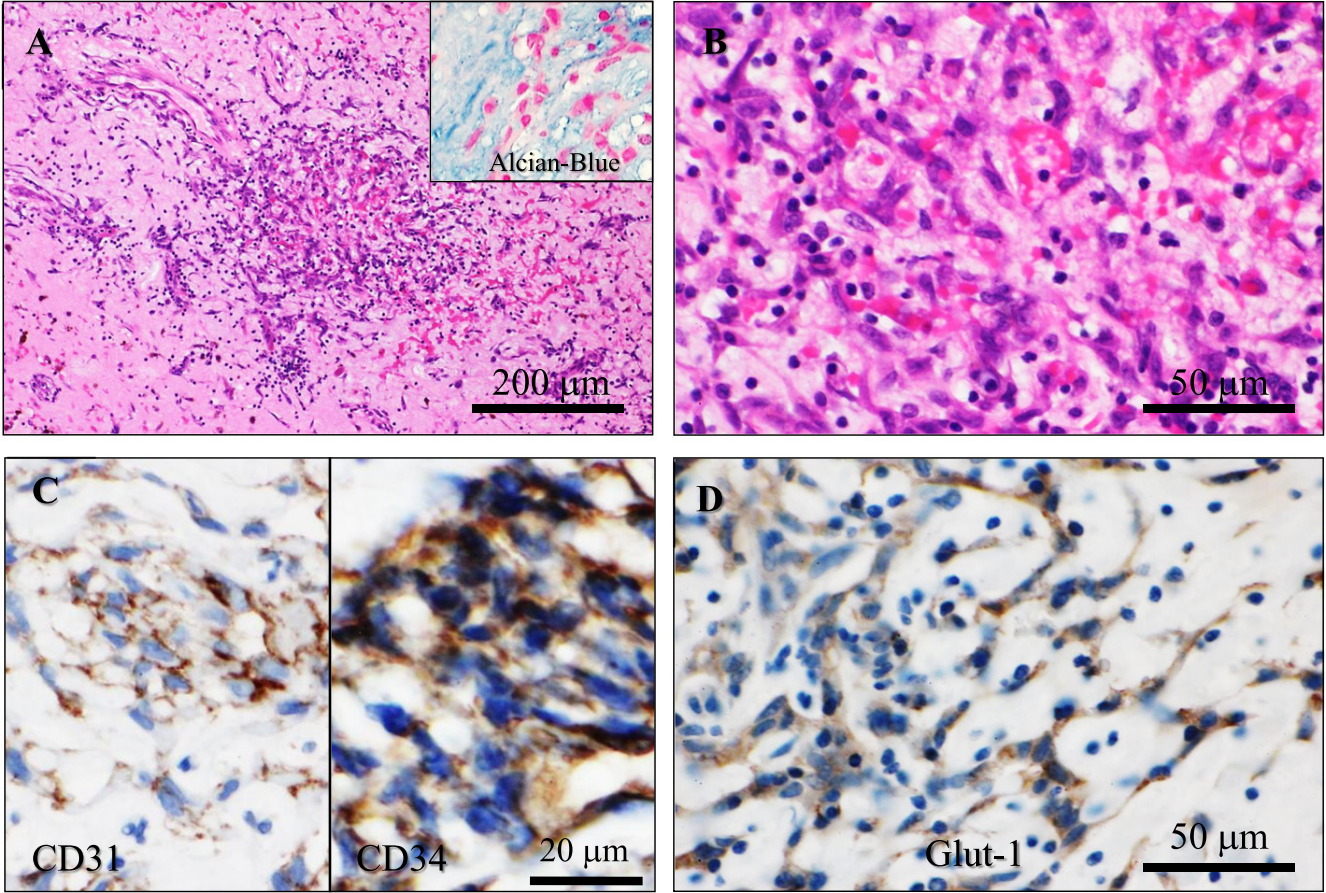
**Microscopic and immunohistochemical examination of the left axial myxoma. (A)** Medium power view showed an acellular to partly cellular proliferation of spindled tumour cells without significant atypia, arranged in variably ring-like structures surrounding small blood vessels with a lymphoplasmacytic infiltrate, embedded in a prominent Alcian-Blue-positive myxoid matrix (inset) (H&E stains). Bar = 200 μm. **(B)** On high-power view, the tumour cells had oval to spindle nuclei, eosinophilic cytoplasm and indistinct cell borders, and inconspicuous nucleoli, manifesting as so-called ‘myxoma cells’ and ‘lepidic cells’, appearing as short cords or syncytia (H&E stains). Bar = 50 μm. **(C)** In immunohistochemistry, the myxoma cells were specifically positive for CD31 (lt.) and CD34 (rt.). Bar = 20 μm. **(D)** Interestingly, a substantial number of these myxoma cells were immunoreactive with GLUT-1 in a cytoplasmic and membranous expression pattern. Bar = 50 μm.

The patient presented here, a 61-year-old female with an unremarkable previous medical history, had no specific clinical symptoms for long periods before the diagnosis. Laboratory data, including blood cell count, chemistry and tumour markers, or electrocardiogram (ECG) were also within normal limits. A detailed medical health examination incidentally detected an intracardiac tumour lesion by a combined ^18^ F-FDG PET/CT scan. Coronal maximum intensity projection (Figure 1A) and axial (Figure 1B) images in coregistered ^18^ F-FDG PET/CT showed a large and mildly to moderately hypermetabolic area in the left atrium (maximal standardized uptake value (SUV): 3.0), which corresponded to a hypodensisty mass lesion on chest CT (Figure 1C), measuring 46 × 32 mm in diameter. Subsequent transthoracic echocardiography (Figure 1D) demonstrated a pedunculated mass originating from the interatrial septum, highly suggestive of left atrial myxoma. Moreover, the neck, chest, and abdomen disclosed no definite evidence of tumour lesions, such as metastatic foci in the lymph nodes or other organs. Surgeons also considered to be a benign intracardiac myxoma and performed a simple excision with repair of the resulting septal defect by a pericardial patch. On gross examination, a gelatinous tumour with a relatively smooth surface was attached to the fossa ovaris by a narrow stalk, and there were no organized thrombi on the surface. Its cut surface showed a well-circumscribed, encapsulated and variegated mass, measuring approximately 40 × 35 mm in diameter, which looked hemorrhagic in color and displayed a gelatinous appearance with gritty calcified areas. Microscopic findings demonstrated an acellular to partly cellular proliferation of spindled tumour cells without significant atypia, arranged in variably ring-like structures surrounding small blood vessels with a lymphoplasmacytic infiltrate, embedded in a prominent Alcian-Blue-positive myxoid matrix with frequent hemorrhage or hemosiderin pigments, and focal hyalinized fibrosis, ossification or calcification (Figure 2A). On high-power view, the tumour cells had oval to spindle nuclei, eosinophilic cytoplasm and indistinct cell borders, and inconspicuous nucleoli, manifesting as so-called ‘myxoma cells’ and ‘lepidic cells’, appearing as short cords or syncytia (Figure 2B). Overall, the main features were consistent with typical cardiac myxoma. In immunohistochemistry [5], those myxoma cells were positive for CD31 (Dako, Glostrup, Denmark, diluted 1:20) and strongly positive for CD34 (Immuno Tech. Co., Ltd., Osaka, Japan, diluted 1:150) (Figure 2C). Interestingly, a substantial number of them were immunoreactive with GLUT-1 (Dako, diluted 1:600) in a cytoplasmic and membranous expression pattern (Figure 2D). In contrast, they were completely negative for cytokeratin (AE1/AE3; Dako, diluted 1:500), CD68 (KP-1; Dako, diluted 1:100), Podoplanin (D2-40; Nichirei Bioscience Co., Tokyo, Japan; diluted 1:1), α-SMA (Dako, diluted 1:500), desmin (Dako, diluted 1:300), or S-100 protein (Dako, diluted 1:900). The MIB-1 labeling index (Ki67; Dako, diluted 1:50) was noted in less than 1 to 3% in the myxoma cells. Based on all these features, we finally made a diagnosis of left atrial myxoma. To date, approximately 1 year routine follow-up after the surgery is established, and the patient remains well and no recurrence has been identified.

Indeed, myxomas, as many as 60% of relatively rare cardiac tumours, could be considered to be common diseases, compared with some recently published papers of very unusual tumour cell types or features in the heart [2,6]. Despite that, merely three case reports of atrial myxoma have demonstrated its appearance and utility in ^18^ F-FDG PET, revealing a moderately elevated glucose metabolism, very similar to our case, and likely assuming an established position in the routine clinical evaluation of cardiac tumours [2-4]. Furthermore, we describe the present unusual case, since it is conceivable that the current short report of cardiac myxoma located on the left atrium is clinicopathologically remarkable for another reason at least. A higher ^18^ F-FDG uptake rate in the present case could have a close correlation with a more specific and greater immunohistochemical expression of GLUT-1 in the myxoma cells, corresponding to the malignant cells in intraductal papillary mucinous neoplasms of the pancreas, as previously reported [7]. Enhanced expression of GLUT-1 has actually been recognized in various cancer tissues examined, since one of the pivotal functions of ubiquitous GLUT-1 is known to particularly increase the glucose supply to dividing and growing cells in part, among a family of GLUT transporters [8,9], possibly unlike these benign myxoma cells with relatively low MIB-1 index. However, the potential biological roles of accelerated glucose utilization in neoplastic cells still remain to be elucidated in detail. Nevertheless, it would be intriguing to assess the significance of ^18^ F-FDG PET findings for cardiac myxoma and the association with immunohistochemical GLUT-1 expression in the myxoma cells on future larger studies. The present short case report could interest the scientific community, taken together with potentially new and specific clinicopathological findings.

### Consent

Written informed consent was obtained from the patient for the publication of this report and any accompanying images.

## Competing interests

The authors declare that they have no competing interests.

## Authors’ contributions

SY and YO participated in conception of the idea and writing of the manuscript. SY, YO, SK, IM, EN, TW, YS, YH and TI performed the clinical imaging and pathological/immunohistochemical interpretation of the tumor tissue. All authors have read and approved the final manuscript.
